# Coverage Probability and Area Spectral Efficiency of Clustered Linear Unmanned Vehicle Sensor Networks

**DOI:** 10.3390/s17112550

**Published:** 2017-11-05

**Authors:** Haejoon Jung, In-Ho Lee

**Affiliations:** 1 Department of Information and Telecommunication Engineering, Incheon National University, Incheon 22012, Korea; haejoonjung@inu.ac.kr; 2 Department of Electrical, Electronic and Control Engineering, Hankyong National University, Anseong 17579, Korea

**Keywords:** linear sensor networks, unmanned vehicle, cluster process, coverage, spectral efficiency

## Abstract

In this paper, we consider clustered unmanned vehicle (UV) sensor networks for swarm sensing applications in a linear structure such as highway, tunnel, underwater pipelines, power lines, and international border. We assume that the linear UV sensor networks follow Thomas cluster process (TCP), in which the cluster locations are modelled by Poisson point process (PPP), while the cluster members (UVs) are normally distributed around their cluster centers. We focus on communications between UVs within a cluster such as local sensing data transfer or swarm coordination, where multiple UV pairs can share the same frequency band simultaneously. Thus, in the presence of co-channel interference both from the same cluster and the other clusters, we study the coverage and area spectral efficiency of the clustered UV sensor networks in a linear topology.

## 1. Introduction

Robotic systems have brought significant benefits to human lives over the past few decades [1]. To extend the functional range of the robotic systems or to deploy them in unstructured environments, robotic technologies are integrated with communication technologies, fostering the emergence of networked robotics [2,3,4,5]. In the networked robotics applications, multiple robots perform a team task in a distributed fashion by exchanging sensing data via the communication network. For example, a team of networked robots can conduct search and rescue missions in extreme environments, such as the earthquake, exploring the unknown space, operating fast and accurate grasp of the real demand as highlighted in [6,7]. The conventional pre-programmed robots cannot be used in these scenarios because of the unknown conditions and time-varying characteristics.

In this paper, we are interested in the applications of networked robotics for monitoring and emergency management in linear structures such as tunnel, pipelines, subway, power lines, and international border. In particular, unmanned vehicles (UVs), which can travel through a long pipe or tunnel-like systems, are useful for search and rescue applications in uncertain disaster environments such as chemical subway attack, nuclear explosion, and fire in pipelines. To explore these inaccessible environments, it is expected to organize swarms (clusters) of small unmanned ground, water, and airborne vehicles and launch complex missions that comprise several such teams [8]. Focusing on robust operation and cooperative sensing tasks in real time, UVs decompose and allocate tasks using onboard computation and inter-vehicle communication.

### 1.1. Motivation and Related Work

As unmanned vehicles (UVs) become more sophisticated and widely deployed, there is an increasing need for communication systems that allow users to effectively utilize a team of UVs to gather required information [9]. However, to our knowledge, in spite of increasing interest in autonomous sensing applications using UVs, there have not been systematic modelling and analysis of UV sensing networks organized as multiple clusters. In particular, how to optimize a random linear or one-dimensional (1D) network with clustering property has not been extensively studied. For example, the prior works in [10,11] considered cooperative multi-hop linear networks with a fixed (deterministic) inter-node separation. In addition, in [12,13]. the impact of intra-flow interference was studied in multi-hop linear networks using continuum assumption, which does not account for random node locations with clustering. Thus, motivated by the lack of a systematic study of *randomly clustered* linear UV networks, in this paper, we present a stochastic geometry-based linear UV network model and provide analytical framework for efficient communication to feed sensed data and team operation in linear UV sensor networks using stochastic geometry.

Stochastic geometry is an effective tool to analyze wireless networks with random topology in a statistical fashion. The main strength of the stochastic geometry-based network modelling is to capture the spatial randomness inherent in wireless networks [14,15]. Moreover, it is straightforward to incorporate with random propagation impairments such as fading, shadowing, and power control [16]. In addition, it often leads to closed-form expressions or bounds that characterize how a large-scale network behaves as key system parameters change. In particular, in interference-limited networks with high node density, stochastic geometry is a powerful technique to simplify modelling and provide accurate enough insights into various wireless networks [17].

Most of the existing work using stochastic geometry focused on ad hoc and sensor networks to account for their intrinsic spatial randomness in the absence of infrastructure [18,19,20,21,22]. On the other hand, because cellular networks were known to be deployed according to an idealized hexagonal grid, stochastic geometry was not widely used to model cellular networks until the early 2010s [16]. However, since it was highlighted that cellular networks also follow an irregular topology, which randomly changes from one geographical location to another, in [23,24], stochastic geometry has attracted significant attention to model and analyze cellular networks. For example, heterogeneous cellular networks were studied in [25,26,27,28,29,30]. In addition, cognitive and self-organizing cellular networks were analyzed in [31,32]. The extensions to coordinated multipoint (CoMP) were characterized in [33,34].

In stochastic geometry analysis, the networks are abstracted to a point process (PP) that reflects characteristics of a given network. Because of its tractability, Poisson point process (PPP), in which the number of points inside any compact set is a Poisson random variable and the points are uniformly distributed in the compact set, is the most commonly used PP. However, PPP cannot capture clustering and repulsion behaviors exhibited by certain network systems such as sensor networks and mobile social networks. For this reason, in recent studies, more complex but more accurate PPs have been employed to better model different types of networks. For instance, in [35,36] Ginibre process was adopted to consider repulsion among nodes (points) in energy harvesting networks. On the other hand, to reflect the clustering and social nature of device-to-device (D2D) networks, in [37,38], clustered D2D networks were studied assuming Thomas cluster process (TCP), where cluster centers are modelled by Poisson point process (PPP) and the cluster members are normally distributed around their cluster centers, in two-dimensional (2D) and three-dimensional (3D) space, respectively.

When it comes to UV sensor networks to monitor spatial phenomena, Gaussian deployment is widely adopted [39], by which the networks exhibit clustering characteristics. Furthermore, it has been shown that wireless nodes have the clustering property in linear vehicular networks with autonomous robotics and driverless vehicle technologies. In [40,41,42], it was presented that the social properties of vehicular ad hoc networks (VANETs) based on trajectory data collected in urban environments. Moreover, in [43], it was shown that the node degree (or node density) distribution of VANET can be modelled by Gaussian distribution in both urban and highway environments. In [44], it was showed that, in VANETs for highway environments, the degree distribution is Gaussian with a high clustering coefficient.

Therefore, considering the node locations following Gaussian distribution and the clustering property, TCP is employed to analyze linear UV sensor networks in this paper. In addition, to optimize the network performance, we adopt the analytic approach in [37,38], because UVs can be regarded as clustered mobile devices in D2D networks in a broad sense. However, it is noted that the statistical analysis of communication distance in 1D space is definitely different from those in 2D and 3D spaces, and the analytic expressions presented in this paper are not the special cases of those derived in [37,38]. As the studies on unmanned aerial vehicle (UAV) networks in [45,46,47], we assume UVs randomly move but transmit only when they are static. In other words, the channel is assumed to be quasi-static, where the network topology may vary over time, but the symbol duration is significantly smaller than the coherence time of the channel, meaning that the topology and fading channel remain the same over an entire symbol period. This assumption perhaps cannot be applied to highly mobile UV networks, but the system performance obtained under this assumption may be used as an upper bound on that of the UV network with high mobility since link reliability can be degraded by high mobility of nodes.

### 1.2. Originalities and Contributions

The original contribution of our work is to present the stochastic geometry-based analysis of 1D clustered UV sensor networks using TCP. The detailed contributions of this paper are four-fold. First, we derive the probability distributions of distance between two UVs in the same cluster and two different clusters, respectively, in the 1D clustered network. Second, we provide the exact mathematical expressions of the coverage probability and the area spectral efficiency. Third, the approximate upper and lower bounds of the coverage probability are obtained, which are useful to gain design insights to improve coverage. Lastly, we present numerical results, which validate our analysis and show the impacts of system parameters.

### 1.3. Organization

The rest of this paper is organized as follows: In Section 2, we introduce our system model of clustered linear UV networks. In Section 3, we derive the probability distributions of the distances between UVs that belong to the same and different clusters. In Section 4, we analyze the network performance in terms of coverage probability and area spectral efficiency. Then, in Section 5, upper and lower bounds of the coverage probability are provided. Section 6 presents numerical results to validate our analysis by comparing with simulation results. The final section concludes the paper and shows some future perspectives.

## 2. System Model

**Figure 1:**
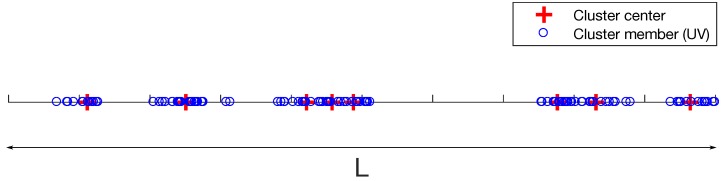
An example illustration of an one-dimensional clustered linear unmanned vehicle (UV) sensor network based on Thomas cluster process (TCP).

We consider a UV sensor network in one-dimensional space with the length of *L* as shown in Figure 1, where the UVs are distributed in clusters. In other words, there exist multiple clusters for the swarm sensing applications, each of which consists of a group of UVs. We assume that each UV communicates with other UVs in the same cluster, while the UVs across clusters do not communicate directly. The locations of the UVs in the 1D linear space are modelled by a TCP, where the cluster centers follow a homogeneous Poisson point process (PPP) $\Phi_{c}$ with density $\lambda_{c}$. In addition, the cluster members (UVs) are independent and identically distributed (i.i.d.) according to a symmetric normal distribution with variance $\sigma^{2}$ around each cluster center $x\in \Phi_{c}$ with the Gaussian density function of the UV locations y∈R relative to a cluster center as

(1) $$f_{Y}\left(y\right)=\frac{1}{\sqrt{2\pi \sigma^{2}}}\exp\left(-\frac{y^{2}}{2\sigma^{2}}\right),$$

where σ is the scattering parameter.

The UVs in the cluster of $x\in \Phi_{c}$ are denoted by $N^{x}$, which has two subsets: (i) transmitting UVs $N_{t}^{x}$; and (ii) receiving UVs $N_{r}^{x}$. Suppose the set of simultaneously transmitting UVs in the cluster is $B^{x}\subseteq N_{t}^{x}$, and its cardinality $|B^{x}|$ follows a Poisson distribution with mean $\lambda_{t}$. In other words, the number of simultaneously active UV transmitters (UV-Txs) inside each cluster is a Poisson random variable with mean $\lambda_{t}$. As in [37], without loss of generality, we analyze based on a typical UV in a representative cluster $x_{0}\in \Phi_{c}$, where the typical UV is regarded as the UV receiver of interest. We assume that the typical UV is located at the origin. In addition, the UVs only transmit while they are static, as in [45].

We assume that the serving (or desired) UV-Tx is located at $y_{0}$ inside the cluster $x_{0}\in \Phi_{c}$. Thus, the distance between the serving UV-Tx and the typical UV is denoted by $r=|y_{0}+x_{0}|$. Hence, with the same transmit power of the UVs denoted by $P_{u}$, the received power at the typical UV is

(2) $$S=\frac{P_{u}h_{0}}{r^{\alpha }}=\frac{P_{u}h_{0}}{|x_{0}+y_{0}{|}^{\alpha }},$$

where α is the path-loss exponent and $h_{0}$ is the power gain of small scale fading channel, which follows exponential distribution with unit mean as in [4,37,48]. The typical UV suffers from two types of co-channel interference: (i) intra-cluster interference caused by the simultaneously active UV-Txs in the same cluster; and (ii) inter-cluster interference caused by the UV-Txs in the other clusters, which are represented as

(3) $$\begin{array}{c} I_{intra}=\sum_{y\in B^{x_{0}}\backslash y_{0}}\frac{P_{u}h_{y_{x_{0}}}}{|x_{0}{+y|}^{\alpha }}, \end{array}$$

(4) $$\begin{array}{c} I_{inter}=\sum_{x\in \Phi_{c}\backslash x_{0}}\sum_{y\in B^{x}}\frac{P_{u}h_{y_{x}}}{{\left|x+y\right|}^{\alpha }}, \end{array}$$

respectively. Consequently, assuming interference-limited networks, the signal-to-interference-ratio (SIR) at the typical UV is given by

(5) $$\mathrm{SIR}(r)=\frac{S}{I_{intra}+I_{inter}}.$$

Since $P_{u}$ is cancelled, we can set $P_{u}=1$ in the SIR analysis.

## 3. Communication Distance Distributions

In this section, we derive the probability distributions of the distances from the typical UV to intra-cluster and inter-cluster UVs for system performance analysis associated with SIR. We assume that the content (or data) of interest for a typical UV in a given cluster is available at a UV chosen uniformly at random in the cluster, as in [37]. Based on this assumption, we derive the distance distributions from the typical UV to the serving UV-Tx, intra-cluster and inter-cluster interferers.

### 3.1. Distance between Typical UV and Intra-Cluster UV-Tx

For the intra-cluster UVs, let $D_{t}^{x_{0}}$ be the set ${\left\{D_{i}\right\}}_{i=1:|N_{t}^{x_{0}}|}$ of distances from the typical UV to the set of possible transmitting UVs $N_{t}^{x_{0}}$ in the cluster $x_{0}\in \Phi_{c}$, where $d_{i}=\left|x_{0}+y\right|$ is the realization of $D_{i}$. We note that the index *i* will be omitted when it is clear from the context. First, if we denote the distance between the cluster center and the typical UV by $v_{0}=\left|x_{0}\right|$, its probability density function (PDF) is the folded normal distribution as

(6) $$f_{v_{0}}\left(v_{0}\right)=\sqrt{\frac{2}{\pi \sigma^{2}}}\exp\left(-\frac{v_{0}^{2}}{2\sigma^{2}}\right),$$

where $v_{0}\geq 0$. In addition, the locations of the cluster center $x_{0}$ and the UV-Txs *y* are i.i.d. random variables in R, following i.i.d. Gaussian distributions with zero mean and variance of $\sigma^{2}$. Thus, $D=|x_{0}+y|$, which is the absolute value of a Gaussian random variable with zero mean and variance of $2\sigma^{2}$, follows a folded normal distribution [49] with the PDF as

(7) $$f_{D}\left(d\right)=\frac{1}{\sqrt{\pi \sigma^{2}}}\exp\left(-\frac{d^{2}}{4\sigma^{2}}\right),$$

where d≥0.

### 3.2. Conditional Distance Distribution given $|x_{0}|$

The distances between the typical UV to the transmitting UVs in the same clusters, which are required to calculate *S* and $I_{intra}$ in SIR, are correlated because of the common factor $x_{0}$. Therefore, conditioning the relative location of the cluster center, $x_{0}$, to typical UV, we can treat the locations of the intra-cluster UVs as i.i.d. random variables, which means that the distances between typical UV and the intra-cluster UVs are i.i.d.

Conditioned on $v_{0}=\left|x_{0}\right|$, $D=|x_{0}+y|$ is the absolute value of a Gaussian random variable with mean of $v_{0}$ and variance of $\sigma^{2}$. Therefore, *D* also follows a folded normal distribution with the PDF as

(8) $$f_{D}\left(d|v_{0}\right)=\sqrt{\frac{2}{\pi \sigma^{2}}}\exp\left(-\frac{d^{2}+v_{0}^{2}}{2\sigma^{2}}\right)\cosh\left(\frac{v_{0}d}{\sigma^{2}}\right),$$

where d≥0 and cosh(·) is the hyperbolic cosine function.

### 3.3. Distances to Serving UV-Tx and Interferers: r, w, and u

Let the serving and intra-cluster interferer distances be $r=|x_{0}+y_{0}|$ and $w=|x_{0}+y|$, respectively. Their conditional PDFs given that $v_{0}=\left|x_{0}\right|$ are the same as Equation (8). In other words, $f_{R}\left(r|v_{0}\right)=f_{D}\left(r|v_{0}\right)$ and $f_{W}\left(w|v_{0}\right)=f_{D}\left(w|v_{0}\right)$. In addition, conditioned on the distance v=|x| between one of the other clusters $x\in \Phi_{c}$ and the typical UV, the distances $\left\{u=|x+y|,\forall y\in B^{x}\right\}$ between the typical UV and the inter-cluster interfering UV-Txs in $x\in \Phi_{c}$ are i.i.d., following the conditional PDF $f_{U}\left(u|v\right)=f_{D}\left(u|v_{0}=v\right)$ given in (8).

### 3.4. Validation through Simulation

**Figure 2:**
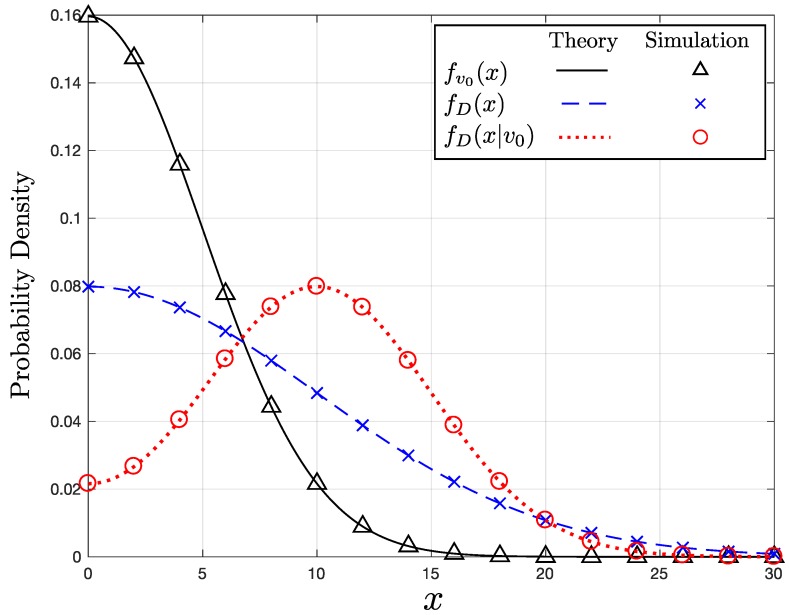
Example PDFs with σ=5.

Figure 2 shows the three PDFs: $f_{v_{0}}\left(x\right)$, $f_{D}\left(x\right)$, and $f_{D}\left(x|v_{0}\right)$ in Equations (6)–(8), respectively. For the conditional PDF of *D*, $f_{D}\left(x|v_{0}\right)$, we set $v_{0}=10$. The solid, dotted, and dashed lines represent the theoretical graphs based on the derived PDFs. On the other hand, the three differently shaped markers indicate the corresponding simulation results. As shown in the figure, the analytical results are consistent with the simulation results for the entire range of *x*, which verifies our analysis.

## 4. Performance Analysis

In this section, we investigate the coverage probability, denoted by P_c_, and the area spectral efficiency, denoted by ASE, of the 1D clustered UV sensor network. We first find the Laplace transforms of the two interference terms to characterize SIR. Then, using the Laplace transforms, we derive the exact expressions of P_c_ and ASE.

### 4.1. Laplace Transform of Intra-Cluster Interference

Conditioned on $v_{0}=\left|x_{0}\right|$, we first derive the Laplace transform of $I_{intra}$ as

(9) $$\begin{array}{cc} L_{I_{intra}}\left(s|v_{0}\right) & =E\left[e^{-sI_{intra}}\right]\\ & =E_{B^{x}}\left[\prod_{y\in B^{x}}E_{h_{y_{x_{0}}}}\left[\exp\left(\frac{-sh_{y_{x_{0}}}}{{\left|x+y\right|}^{\alpha }}\right)\right]\right]\\ & \overset{\left(a\right)}{=}E_{B^{x_{0}}}\left[\prod_{y\in B^{x_{0}}\backslash y_{0}}\frac{1}{1+s|y+x_{0}{|}^{-\alpha }}\right]\\ & \overset{\left(b\right)}{=}\exp\left(\left(1-\lambda_{t}\right)\int_{R}\frac{s|y+x_{0}{|}^{-\alpha }}{1+s|y+x_{0}{|}^{-\alpha }}f_{Y}\left(y\right)dy\right)\\ & \overset{\left(c\right)}{=}\exp\left(\left(1-\lambda_{t}\right)\int_{0}^{\infty }\frac{sw^{-\alpha }}{1+sw^{-\alpha }}f_{W}\left(w|v_{0}\right)dw\right), \end{array}$$

where (a) follows from the exponentially distributed $h_{x_{0}}$ with unit mean, and (b) follows from the probability generating functional (PGF) of Poisson process [50]. In addition, (c) follows from $w=|x_{0}+y|$.

### 4.2. Laplace Transform of Inter-Cluster Interference

The Laplace transform of $I_{inter}$ is given by

(10) $$\begin{array}{cc} L_{I_{inter}}\left(s\right) & =E\left[e^{-sI_{inter}}\right]\\ & =E_{\Phi_{c}}\left[\prod_{x\in \Phi_{c}\backslash x_{0}}E_{B^{x}}\left[\prod_{y\in B^{x}}E_{h_{y_{x}}}\left[\exp\left(\frac{-sh_{y_{x}}}{{\left|x+y\right|}^{\alpha }}\right)\right]\right]\right]\\ & \overset{\left(a\right)}{=}E_{\Phi_{c}}\left[\prod_{x\in \Phi_{c}\backslash x_{0}}E_{B^{x}}\left[\prod_{y\in B^{x}}\frac{1}{{1+s|y+x|}^{-\alpha }}\right]\right]\\ & \overset{\left(b\right)}{=}E_{\Phi_{c}}\left[\prod_{x\in \Phi_{c}\backslash x_{0}}\exp\left(\int_{0}^{\infty }\frac{-\lambda_{t}su^{-\alpha }}{1+su^{-\alpha }}f_{U}\left(u|v\right)du\right)\right]\\ & \overset{\left(c\right)}{=}\exp\left(2\lambda_{c}\int_{0}^{\infty }\left(\kappa (v)-1\right)dv\right), \end{array}$$

where $\kappa \left(v\right)=\exp\left(\int_{0}^{\infty }\frac{-\lambda_{t}su^{-\alpha }}{1+su^{-\alpha }}f_{U}\left(u|v\right)du\right)$ and (a) follows from the exponentially distributed $h_{x_{0}}$ with unit mean. In addition, (b) and (c) follow from the PGF of Poisson process.

### 4.3. Coverage Probability and Area Spectral Efficiency

Letting β denote the SIR threshold for successful decoding at the receiver, which is a function of modulation and coding, the coverage probability is

$$\begin{array}{cc} P_{c} & =P[\mathrm{SIR}>\beta ]=E_{R}\left\{P[\mathrm{SIR}(R)>\beta |R]\right]\}\\ & =E_{R}\left\{P[h_{0}>\beta r^{\alpha }\left(I_{intra}+I_{inter}\right)|R=r]\right\}\\ & =E_{R}\left\{E\left\{e^{-\beta r^{\alpha }\left(I_{intra}+I_{inter}\right)}|R=r\right\}\right\}\\ & =\int_{0}^{\infty }\int_{0}^{\infty }L_{I_{inter}}\left(\beta r^{\alpha }\right)L_{I_{inter}}\left(\beta r^{\alpha }|v_{0}\right)f_{R}\left(r|v_{0}\right)f_{v_{0}}\left(v_{0}\right)drdv_{0}. \end{array}$$

Therefore, letting the area spectral efficiency be defined as the average achievable rate per unit bandwidth per unit area as in [37], the area spectral efficiency is given

(11) $$\begin{array}{c} \mathrm{ASE}=\lambda_{t}\lambda_{c}\log_{2}\left(1+\beta \right)P_{c}, \end{array}$$

where $\lambda_{t}\lambda_{c}$ is the average density of simultaneously active UV-Txs of the whole UV sensor network.

## 5. Approximate Upper and Lower Bounds of P_c_

As the exact expressions of P_c_ and ASE are unwieldy, we provide easy-to-compute upper and lower bounds of P_c_. Particularly, the lower bound is in a closed form, which can be readily evaluated. As stated in Section 2, *r* and *w* are correlated because of the common factor $x_{0}$. For analytical tractability, to derive the two approximate bounds we allow separate de-conditioning on *r* and *w* as in [37], which implies that *r* and *w* are treated as i.i.d. random variables following the PDF in (7).

### 5.1. Upper Bound of *P_c_*

Since the intra-cluster interferers are significantly closer to the typical UV compared to the inter-cluster UV-Txs, $I_{intra}$ is dominant in the denominator of SIR. Thus, we can derive the approximate upper bound of SIR by ignoring $I_{inter}$, which corresponds to the upper bound of P_c_. By the i.i.d. assumption of *r* and *w*, the Laplace transform of $I_{intra}$ can be approximated as

(12) $${\tilde{L}}_{I_{intra}}\left(s\right)=e^{\left(1-\lambda_{t}\right)\int_{0}^{\infty }\frac{sw^{-\alpha }}{1+sw^{-\alpha }}f_{W}\left(w\right)dw},$$

where $f_{W}\left(w\right)$ follows the PDF in Equation (7). Thus, the upper bound of P_c_ is given by

(13) $$\begin{array}{cc} \tilde{P_{c}} & =E_{R}\left\{P[h_{0}>\beta r^{\alpha }I_{intra}|R=r]\right\}\\ & =\int_{0}^{\infty }{\tilde{L}}_{I_{intra}}\left(\beta r^{\alpha }\right)f_{R}\left(r\right)dr, \end{array}$$

where $f_{R}\left(r\right)$ follows the PDF in Equation (7).

### 5.2. Lower Bound of *P_c_*

We first derive lower bounds of $L_{I_{inter}}\left(s\right)$ and $L_{I_{inter}}\left(s\right)$ in closed forms. Then, using the two, the lower bound of P_c_ will be obtained.

**Corollary** **1.**  *The lower bound on the Laplace transform of $I_{intra}$ is*

(14) $$\begin{array}{c} L_{I_{inter}}\left(s\right)\geq L_{I_{intra}}^{*}\left(s\right)\overset{}{=}\exp\left(\frac{1-\lambda_{t}}{\sigma \sqrt{\pi }}\frac{s^{1/\alpha }\left(\pi /\alpha \right)}{\sin(\pi /\alpha )}\right) \end{array}$$

**Proof.**  See Appendix A. ☐

**Corollary** **2.**  *The lower bound on the Laplace transform of $I_{inter}$ is*

(15) $$\begin{array}{c} L_{I_{inter}}\left(s\right)\geq L_{I_{inter}}^{*}\left(s\right)\overset{}{=}\exp\left(-2s^{\frac{1}{\alpha }}\frac{\lambda_{c}\lambda_{t}\left(\pi /\alpha \right)}{\sin(\pi /\alpha )}\right). \end{array}$$

**Proof.**  See Appendix B. ☐

Based on Equations (14) and (15) along with the independent de-conditioning assumption, we can obtain the approximate lower bound of P_c_ in a closed form as follows:

(16) $$\begin{array}{cc} P_{c} & \geq \int_{0}^{\infty }L_{I_{inter}}^{*}\left(\beta r^{\alpha }\right)L_{I_{intra}}^{*}\left(\beta r^{\alpha }\right)f_{R}\left(r\right)dr\\ & \overset{\left(a\right)}{=}\int_{0}^{\infty }\exp\left(-\rho r\right)\sqrt{\frac{1}{\pi \sigma^{2}}}\exp\left(-\frac{r^{2}}{4\sigma^{2}}\right)dr,\\ & \overset{\left(b\right)}{=}\exp\left(\rho^{2}\sigma^{2}\right)\left[1-(\rho \sigma )\right]:={P_{c}}^{*}, \end{array}$$

where $\rho =\frac{\pi \beta^{1/\alpha }}{\alpha \sin(\pi /\alpha )}\left(\frac{\lambda_{t}-1}{\sigma \sqrt{\pi }}+2\lambda_{c}\lambda_{t}\right)$ and erf$\left(x\right)=\frac{1}{\sqrt{\pi }}\int_{0}^{x}e^{-t^{2}}dt$ is the error function. Furthermore, (a) follows from $f_{R}\left(r\right)$ in (7) and (b) follows from ρ≥0 (because α≥2 and $\lambda_{t}\geq 1$).

## 6. Numerical and Simulation Results

In this section, we present numerical and simulation results to validate our analysis and discuss the impacts of system parameters on P_c_ and ASE. For simulations, the UV locations are randomly drawn from a TCP over a linear network with L=10 km. The simulation results are obtained by averaging over $10^{6}$ iterations. For each realization, the network topology is randomly generated. To be specific, the cluster centers follow PPP with intensity $\lambda_{c}$, where the average number clusters is $L\lambda_{c}=10\lambda_{c}$ in each run. Furthermore, UVs are randomly located following 1D Gaussian distribution around the cluster centers. Assuming the average number of active UV Txs of $\lambda_{t}$, the average number of UVs in the network in each simulation trial is $L\lambda_{c}\lambda_{t}=10\lambda_{c}\lambda_{t}$. In addition, we assume the path-loss exponent α of 4 as in [37], and the fading channel is independently realized following Rayleigh distribution for any pair of UVs in each simulation trial. To validate the scalability of the presented model and analysis, we change the average number of active UV Txs $\lambda_{t}$ in each cluster from one to twenty, which corresponds to the average number of active UV Txs in the entire network increases up to $200\lambda_{c}$, and observe how network performances vary.

### 6.1. Upper and Lower Bounds

**Figure 3:**
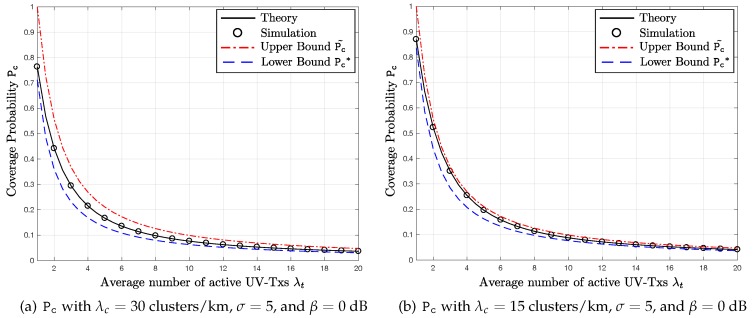
P_c_ versus $\lambda_{t}$: comparison with the upper and lower bounds.

Figure 3 shows the coverage probability P_c_ with different average numbers of simultaneously active UV-Txs $\lambda_{t}$. Figure 3a,b corresponds to $\lambda_{c}=30$ and 15 clusters/km, respectively. For both cases, we use σ=5 and β=0 dB. The solid lines indicate the theoretical results of the coverage probability P_c_, while the circles represent the simulation results. Moreover, the dashed and dash-dotted lines correspond to the lower and upper bounds, $\tilde{P_{c}}$ and ${P_{c}}^{*}$, respectively. As expected, in both Figure 3a,b, all of the curves decrease, as $\lambda_{t}$ increases, because the number of the intra-cluster and inter-cluster interferers increases.

In both figures, comparing the simulation and theoretical curves, we observe the two are consistent to each other, which validates our analysis in the previous sections. Furthermore, the exact coverage probability P_c_ obtained from the simulation results is always bounded by the derived upper and lower bounds $\tilde{P_{c}}$ and ${P_{c}}^{*}$. As expected, when $\lambda_{c}$ is small, which means the lower inter-cluster interference, the upper bound $\tilde{P_{c}}$ is closer to the exact P_c_, where the inter-flow interference is ignored. In contrast, when $\lambda_{c}$ increases from 15 to 30 clusters/km, the lower bound ${P_{c}}^{*}$ becomes closer to the exact P_c_. In addition, as $\lambda_{t}$ rises, both of the upper and lower bounds approach the exact regardless of $\lambda_{c}$.

### 6.2. Impact of $\lambda_{c}$ and σ on $P_{c}$

**Figure 4:**
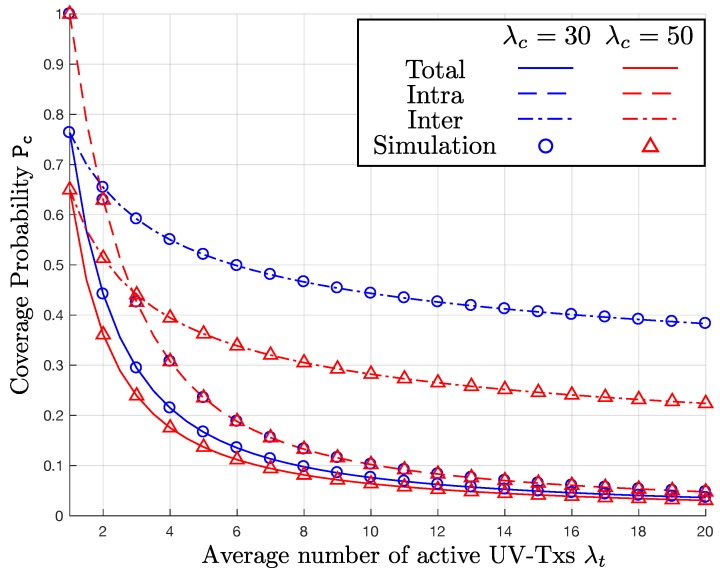
P_c_ versus $\lambda_{t}$ for $\lambda_{c}=\left\{30,50\right\}$ clusters/km, σ=5, and β=0 dB.

**Figure 5:**
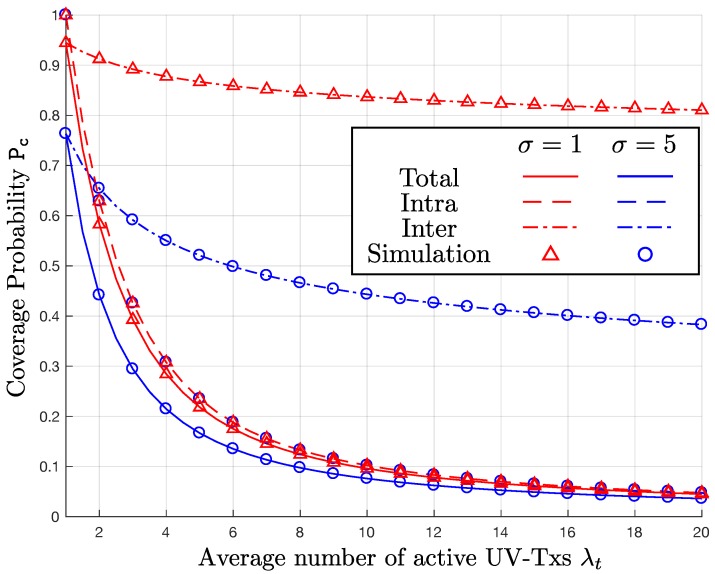
P_c_ versus $\lambda_{t}$ for $\lambda_{c}=30$ clusters/km, σ={1,5}, and β=0 dB.

In Figure 4 and Figure 5, we observe how the exact P_c_ changes with $\lambda_{c}$ and σ. In both figures, the vertical axis is P_c_, while the horizontal axis indicates $\lambda_{t}$. To delve into the impacts of the two parameters, we consider three different coverage probabilities in the presence of: (i) only intra-cluster interference; (ii) only inter-cluster interference; and (iii) both intra-cluster and inter-cluster interference. In the graphs, the three cases correspond to the dashed, dash-dotted, and solid lines, respectively. In addition, the simulation results for $\lambda_{c}=30,50$ are denoted by the “o"- and “x"-markers, respectively.

In Figure 4, the curves only with the intra-cluster interference give the same P_c_ regardless of $\lambda_{c}$, which has nothing to do with the intra-cluster interference. In contrast, as $\lambda_{c}$ increases from 30 to 50 clusters/km, P_c_ obtained only with the inter-cluster interference decreases. As a result, P_c_ based on both the intra- and inter-cluster interference also decreases, as $\lambda_{c}$ increases. In Figure 5, we plot the P_c_ curves with σ=1 and 5. With the two different values of σ={1,5}, the P_c_ curves calculated only with the intra-cluster interference are identical to each other. This is because the variations of the serving and interfering UV-Txs are canceled each other. On the other hand, when σ increases, the coverage probability P_c_ with inter-cluster interference decreases, because of the increased separation from the serving UV-Tx. Consequently, P_c_ with both the intra-cluster and inter-cluster interference also decreases, as σ goes up.

### 6.3. Area Spectral Efficiency

**Figure 6:**
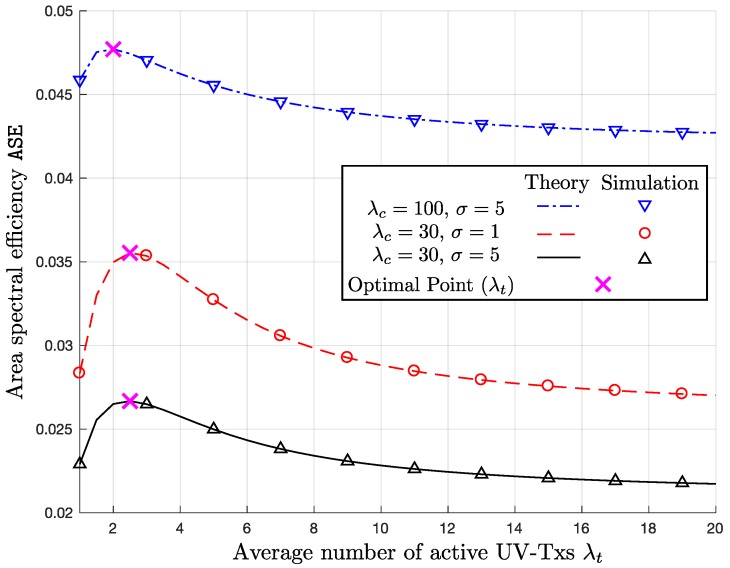
ASE versus $\lambda_{t}$ with $\left(\lambda_{c},\sigma \right)=\{\left(100,5\right)$, (30,1), (30,5)} and β=0 dB.

**Figure 7:**
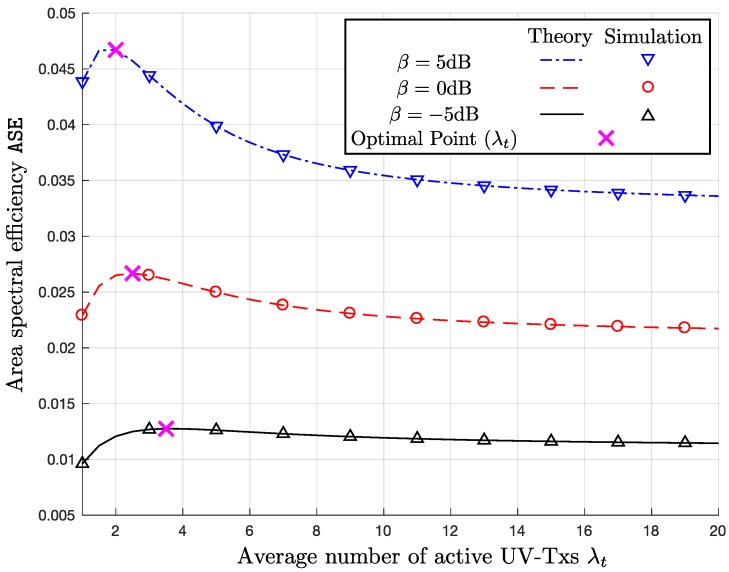
ASE versus $\lambda_{t}$ with $\lambda_{c}=30$ clusters/km, σ=5 and β={−5,0,5} dB.

Figure 6 and Figure 7 show ASE versus $\lambda_{t}$ graphs. In the both figures, the optimal $\lambda_{t}$ to maximize ASE for the given system parameters is indicated by the ‘x’-markers. In Figure 6, we observe the impacts of $\lambda_{c}$ and σ on ASE. If comparing the two curves with $\lambda_{c}=30$ and 100 clusters/km for the same σ=5, ASE increases, as $\lambda_{c}$ increases, because the decrease in P_c_ caused by the increase in $\lambda_{c}$ is not as much as the increase by the multiplicative term of $\lambda_{c}$ in ASE given in (11). On the other hand, as expected from the previous simulation results regarding P_c_, if comparing the two curves with the different σ but the same $\lambda_{c}$, we observe that ASE decreases, as σ increases. If we look at Figure 7, the higher β makes ASE increase, because of $\log_{2}\left(1+\beta \right)$ in (11). Furthermore, the decrease in ASE with β=5 dB is the most rapid, when $\lambda_{t}$ increases, because the higher β implies the tighter SIR requirement.

One of the key design issues is to determine the optimal $\lambda_{t}$, which means how many simultaneously active UV-Txs we should allow. In Figure 6, the changes in σ and $\lambda_{c}$ do not cause significant variation in the optimal $\lambda_{t}\approx 2$. On the other hand, if we decrease the SIR threshold β as in Figure 7, the optimal value of $\lambda_{t}$ increases up to 3.75, because lower β can accommodate more simultaneous UVs.

## 7. Conclusions and Future Work

In this paper, we have studied a clustered linear UV sensor network assuming the search and rescue missions conducted by networked robots in a linear structure. Based on the clustered nature of the swarm sensing applications, we have modelled the linear UV sensor network by TCP, where multiple UVs form a cluster. Using stochastic geometry, we have analyzed P_c_ and ASE of the clustered linear UV sensor network in the presence of co-channel interference both from the same cluster and the other clusters. We have derived the exact mathematical expressions of P_c_ and ASE, which are verified with the simulation results. Moreover, the approximate upper and lower bounds on P_c_ have been derived, which become tighter as $\lambda_{t}$ grows. Both of bounds can provide design insights to achieve a certain level of P_c_. Numerical results indicate that P_c_ can be improved with smaller $\lambda_{c}$ and σ. Furthermore, we have observed that there exists an optimal number of simultaneously active UV-Txs $\lambda_{t}$ that maximizes ASE. A method to find an optimal $\lambda_{t}$ can be further studied for implementation in clustered linear UV networks.

Potential extensions of this paper include addressing a wider scenario with time-variant clustering, inter-cluster communications, different fading channels, and high mobility models. In addition, because all of our contributions in this work are focused on the quasi-static scenario, we will remove this assumption and generalize analysis for high mobility UV networks both for intra-cluster and inter-cluster data transmission in the future. Furthermore, we will improve the analytical model with experimental studies using a large scale testbed as a long-term plan.

## Abbreviations

The following abbreviations are used in this manuscript:

UV	Unmanned vehicle
TCP	Thomas cluster process
PPP	Poisson point process
1D	One-dimensional
2D	Two-dimensional
3D	Three-dimensional
VANETs	Vehicular ad hoc networks
SIR	Signal-to-interference-ratio
PDF	Probability density function
PC	Probability of coverage
ASE	Area spectral efficiency
PGF	Probability generating functional

## Appendix A. Proof of Corollary 1

(A1) $$\begin{array}{c} L_{I_{intra}}\left(s\right)=\int_{R}\exp\left(\int_{R}\frac{\left(1-\lambda_{t}\right)f_{Y}\left(y\right)dy}{1+|y+x_{0}{|}^{\alpha }/s}\right)\cdot f_{Y}\left(x_{0}\right)dx_{0}\\ =\int_{R}\exp\left(\int_{R}\frac{\left(1-\lambda_{t}\right)}{1+{\left|z\right|}^{\alpha }/s}f_{Y}\left(z-x_{0}\right)dz\right)\cdot f_{Y}\left(x_{0}\right)dx_{0}\\ \overset{\left(a\right)}{\geq }\exp\left(\int_{R}\int_{R}\frac{\left(1-\lambda_{t}\right)}{1+{\left|z\right|}^{\alpha }/s}f_{Y}\left(z-x_{0}\right)f_{Y}\left(x_{0}\right)dx_{0}dz\right)\\ \overset{\left(b\right)}{\geq }\exp\left(\int_{R}\frac{\left(1-\lambda_{t}\right)}{1+{\left|z\right|}^{\alpha }/s}{\left|f_{Y}*f_{Y}\right|}_{\infty }dz\right)\\ =\exp\left(\frac{1-\lambda_{t}}{2\sigma \sqrt{\pi }}\int_{R}\frac{1}{1+{\left|z\right|}^{\alpha }/s}dz\right),\\ =\exp\left(\frac{1-\lambda_{t}}{\sigma \sqrt{\pi }}\int_{0}^{\infty }\frac{1}{1+{\left|z\right|}^{\alpha }/s}dz\right),\\ =\exp\left(\frac{1-\lambda_{t}}{\sigma \sqrt{\pi }}s^{1/\alpha }\frac{\left(\pi /\alpha \right)}{\sin(\pi /\alpha )}\right), \end{array}$$

which corresponds to $L_{I_{intra}}^{*}\left(s\right)$ in (14). (a) follows from Jensen’s inequality, and (b) follows from Holder’s inequality.

## Appendix B. Proof of Corollary 2

(A2) $$\begin{array}{cc} L_{I_{inter}} & \left(s\right)\overset{\left(a\right)}{\geq }\exp\left(2\lambda_{c}\;\int_{0}^{\infty }\;\int_{0}^{\infty }\frac{-\lambda_{t}su^{-\alpha }}{1+su^{-\alpha }}f_{U}\left(u|v\right)dudv\right),\\ & \overset{\left(b\right)}{=}\exp\left(-2\lambda_{c}\lambda_{t}\int_{0}^{\infty }\frac{su^{-\alpha }}{1+su^{-\alpha }}dv\right),\\ & =\exp\left(-2s^{\frac{1}{\alpha }}\frac{\lambda_{c}\lambda_{t}\left(\pi /\alpha \right)}{\sin(\pi /\alpha )}\right), \end{array}$$

which gives $L_{I_{inter}}^{*}\left(s\right)$ in (15). (a) follows from the Taylor expansion of an exponential function, and (b) is based on the property of the PDF in (7) that $\int_{0}^{\infty }f_{U}\left(u|v\right)v^{2}dv=u^{2}$.
